# Specific Localization of β-Arrestin2 in Myenteric Plexus of Mouse Gastrointestinal Tract

**DOI:** 10.1371/journal.pone.0103894

**Published:** 2014-08-01

**Authors:** Hercules T. Maguma, Dipanjana Datta De, Sukhada Bhave, William L. Dewey, Hamid I. Akbarali

**Affiliations:** Department of Pharmacology and Toxicology, VCU Program in Enteric Neuromuscular Sciences, Virginia Commonwealth University, Richmond, Virginia, United States of America; Temple University School of Medicine, United States of America

## Abstract

β-arrestin2 is a key molecule involved in signaling and internalization of activated G protein-coupled receptors including µ-opioid receptors (MOR). Previously we have shown that decreased expression of β-arrestin2 upon chronic morphine is associated with the development of opioid tolerance in the gastrointestinal tract. However, the localization of β-arrestin2 within the gastrointestinal wall is not known. In this study we found that β-arrestin2 is localized in the soma of a select group of neurons in the myenteric ganglia but not in smooth muscle. The density of β-arestin2 was significantly higher in the ileum than the colon. We identified four variants of β-arrestin2 in the ileum, with ARRB-005 and ARRB-013 being the most abundant. Further, the current study utilized multiple-labeling immunofluorescence to characterize the chemical coding of neurons expressing β-arrestin2 in the murine myenteric plexus and the co-localization of MOR_1_ and β-arrestin2. β-arrestin2 co-localized with choline acetyltransferase and calretinin. In contrast, β-arrestin2 neither co-localized with substance P, nitric oxide synthase nor calbindin. Genetic deletion of β-arrestin2 did not affect cholinergic neuron activation by nicotine in the isolated ileum (-log M EC_50_: wild type = 5.8 vs. β-arrestin2 knockout = 5.9). Our findings suggest specificity in the localization of β-arrestin2 in the myenteric plexus within MOR_1_-expressing neurons and provide a relation for direct intracellular crosstalk between MOR_1_ receptor activation and β-arrestin2 signaling in the myenteric neurons. β-arrestin2 deletion does not directly alter basal enteric cholinergic neuronal function.

## Introduction

The development of cellular tolerance following activation of G protein-coupled receptors (GPCR) has been attributed in part to the desensitization and internalization of the activated receptor by the class of proteins called arrestins [1], [2]. In particular, β-arrestin2 has been reported to play an integral role as a scaffolding protein for internalization of the GPCR through clathrin-coated pits and subsequent intracellular trafficking to subcellular compartments (e.g. lysosomes) where GPCR degradation or dephosphorylation occurs [3], [4]. In the CNS, where β-arrestin2 has been reported to be ubiquitously expressed [5], it has been shown that genetic deletion of β-arrestin2 attenuates the development of tolerance to opioid's analgesic effect [6]. In contrast, within the colon where morphine tolerance does not manifest, we have demonstrated that the genetic deletion of β-arrestin2 results in the development of tolerance to repeated morphine exposure in isolated colonic circular muscle [7]–[9]. While the reason for the differences between the effect of chronic opioids in the CNS and enteric nervous system (ENS) is not entirely clear, we speculate that this may be due to differential expression environment of this protein in these tissues. We recently demonstrated that chronic opioid exposure resulted in a time-dependent decrease in β-arrestin2 levels in the ileum [8], but do not know whether direct spatial interaction between the endogenous opioid system and β-arrestin2 occurs in the myenteric plexus. According to Taylor and Fleming [10], the criteria for assessing drug induced plasticity must “*quantitatively account for the tolerance*” and “*occur in the very cells upon which the opioid is acting*”. We hypothesize that β-arrestin2 and MOR_1_ receptors are co-localized in the same neuronal cells which provide the basis for intracellular crosstalk that manifests as opioid tolerance. It is upon this basis that we sought to investigate the chemoarchitecture of β-arrestin2 expressing neurons in the myenteric ganglia and whether or not these neurons express MOR_1_ receptors. Moreover, in the NCBI database 13 splice variants of β- arrestin have been reported. Thus, we also elucidated the nature of the variants found in the ileum. Various electrophysiological, neurochemical and morphological techniques have been utilized to characterize myenteric neurons. The most widely recognized morphological classification broadly distinguishes myenteric and submucosal neurons as Dogiel type I or II [11] however, because of the complexity of neuronal structures in larger mammals, more elaborate morphological classifications have been proposed to further distinguish the neurons [12]. An alternative classification criteria involves neurochemical coding of the neurons based on the myriad of neurotransmitters or calcium binding proteins expressed/stored in the cells e.g. substance P, acetylcholine, calretinin etc. [13]. The advantage of neurotransmitter based chemical coding is that it also defines the function of the neuron hence paves the way for functional assessment. The guinea pig longitudinal muscle/myenteric plexus (LMMP) preparation (whole mount) has been widely used to characterize myenteric neurons in part due to its suitability for fine dissection [11], [14], [15]. In this study we chose the murine model cognizant of the difficulty involved in preparing the mouse longitudinal muscle/myenteric plexus (LMMP) and the challenge in assessing neuronal morphological structure since the dendrites are not elaborate [16]. In spite of these drawbacks, the mouse LMMP model has greater utility since one can use genetic knockouts to investigate the functional effect of a specific protein.

In the present study, we established the nature of β-arrestine2 variants found in the ileum by RT-PCR and further utilized dual labeling immunohistochemistry to define the chemoarchitecture of neurons expressing β-arrestin2. We used fluorescence *in situ* hybridization (FISH) to identify MOR_1_ mRNA expression. We report that four of the thirteen splice variants *viz*, ARBB-006, ARBB-005, ARBB-013, ARBB-001 that code for β- arrestin isoforms a and b protein are found in the ileum. Our studies (1) clearly show the intracellular localization of β-arrestin2 and that there is a higher density of β-arrestin2 containing neurons in the ileum than the colon, (2) reveal extensive co-localization between MOR_1_ RNA and β-arrestin2 (3) show selective expression of β-arrestin2 in morphologically homogeneous cholinergic neurons in the myenteric ganglia (4) exclude the expression of detectable levels of β-arrestin2 in the other cell types including glia and longitudinal smooth muscle cells and (5) demonstrate that the abrogation of β-arrestin2 does not directly alter the basal cholinergic neuronal activation.

## Materials and Methods

### Ethics Statement

All experimental procedures using animals were reviewed and approved by the Institutional Animal Care and Use Committee of Virginia Commonwealth University and were conducted in accordance with the guidelines for the humane use of animals in research (NIH “Public Health Service Policy on Humane Care and Use of Laboratory Animals” [revised 2002]).

### Drugs and Chemicals

All drugs were dissolved in distilled water to make 10 µM stock solutions which were further diluted with distilled water to achieve the targeted concentration. Nicotine (as hydrogen tartrate salt), acetylcholine (as chloride salt) and colchicine were procured from Sigma-Aldrich Co. (St. Louis, MO).

### Mice

The breeding pairs for the β-arrestin2 KO mice were obtained from Dr. Lefkowitz (Duke University, Durham, NC) and housed within the transgenic facility at Virginia Commonwealth University. The animals were housed up to five per cage with access to food and water *ad libitum*. The mice were moved to our departmental animal room and kept in the facility for at least one week to permit acclimation prior to the experiments. Every effort was made to reduce the use of animals to the minimum number required to achieve sufficient statistical power.

### Longitudinal muscle/myenteric plexus tissue preparation and staining

Mice were sacrificed by cervical dislocation. Segments of the distal colon (approximately 1 cm from anus) and terminal ileum (2 cm from ileo-cecal junction) were removed and placed in a dissecting dish containing phosphate buffered saline (PBS). The segments of ileum and colon were flushed of their contents and trimmed of mesentery. Ileum or colonic segments (1 cm) were placed in cold PBS solution and cut longitudinally along the mesenteric border. The sheet of tissue was then pinned and stretched on a dissecting plate containing Sylgard with the mucosal side facing up. The tissues were incubated and fixed in 4% paraformaldehyde for 3 hr at room temperature. Following incubation, the paraformaldehyde was removed by rinsing the tissue with PBS. The mucosa, submucosa and circular muscle layers were removed using fine forceps under a dissecting microscope and the LMMP immersed and stored in PBS containing 0.1% sodium azide at 4°C pending immunofluorescence probing. For substance P staining, the excised tissue was initially incubated in Krebs solution bubbled with 95% O_2_ and 5% CO_2_ containing colchicine (0.01 g/100 ml) at 37°C overnight in order to enhance the immunofluorescence [15].

### Real Time PCR

Total RNA was isolated from ileum of C57/BL mouse by TRIZOL method (Invitrogen, Life Technologies, Carlsbad, USA). cDNa was prepared from 5 mg of the total RNA using thermoscript reverse transcriptase (Invitrogen, Life Technologies, Carlsbard, USA). Real time pcr (Biorad,) was performed using sybr green chemistry (Quantifast sybr green, Qiagen,) and primers specific for the isoforms for β- arrestin. The primers used for the four variants are *ARBB-013/ARBB-005 F: 5′-CCCCAGTCAGCGCCCATCCA-3′*, *ARBB-013/ARRB-006 R: 5′- CAAAGTCCTCAAACACGATG-3′*, *ARRB-006/ARRB-001 F: 5′-AGTCAGCCCCCCGGGAAACAGA-3′*,*ARRB-005/ARRB-001 R: 5′-CCTTGGAACACTGGCATAG-3′*. The numbers for the primers indicate the variant it amplifies. For each variant three biological and three technical repeats were performed. The mean Ct value was plotted ± SD.

### Fluorescence *in situ* hybridization procedure (FISH)

#### Probe preparation

Total RNA was extracted from ileum of C57/Bl6 mouse according to the manufacturer's protocol by Trizol method (Invitrogen, Life Technologies, Carlsbad, USA). 10 µg of total RNA was reverse transcribed using a Superscript indirect cDNA labeling kit (Invitrogen, Life Technologies, Carlsbard, USA) and OPRM-1 gene specific primers incorporating Cy5 labeled dCTP (Amersham Biosciences UK) into the synthesized cDNA. The primer pool used to synthesize the gene specific cDNA were designed to amplify the different splice variants. The primer sequences used are FISH cDNA1: 5′-ACTCAAGAGTGTTCGGGTTCC-3′, FISH cDNA 2∶5′-ATGAACATTACGGGCAGACC-3′,FISH cDNA 3∶5′- AGTGTTTTGACGGATTCGAGCAG-3′,FISH cDNA 4∶5′-GCAGTCTTCATTTTGGTATAT-3′. Each primer identifies one or more splice variants of OPRM-1 gene reported in brain, since there is little or no sequence information known about the type of OPRM-1 variant that might be present in the mouse ileum.

#### *In situ* hybridization

Tissues were fixed with 4% paraformaldehyde in PBS for 20 min at room temperature followed by incubation with proteinase K in Tris/EDTA buffer for 30 min. The tissues were then refixed using 4% paraformaldehyde. Whole mounts of LMMP were then pretreated with 0.5% Triton X-100 to solubilize the cell membrane and increase penetration of the probe. Prior to the incubation with the Cy5-labelled oligonucleotide probe, the whole mount was incubated in a pre-hybridization buffer (containing pre-hybrid solution, yeast tRNA, heparin and formamide) for 1 hr. Incubation with the oligonucleotide probe was performed overnight at 65°C. Following overnight incubation the probe was removed using saline-sodium citrate buffer and the sample cover-slipped for imaging.

### Immunofluorescence procedures

#### Immunostaining procedure

Whole mounts of LMMP or isolated cells were pretreated with 0.5% Triton X-100 to solubilize the cell membrane and increase penetration of the primary antibodies. Prior to primary antibody incubation, the whole mount or isolated cells were blocked with either goat (10%) or donkey serum (10%) for 45 minutes. Dual-labeling experiments were performed with simultaneous incubation of primary antibodies raised in different species targeting the proteins of interest. The tissues were incubated in PBS containing the primary antibodies overnight at 4°C. Following the overnight incubation, the tissues/cells were washed with PBS and then incubated for 4 hours at room temperature in a mixture containing the secondary antibodies. Negative control experiments excluded the primary antibodies and these revealed negligible faint labeling due to non-specific binding of the secondary antibodies. Following incubation with the respective secondary antibodies, the LMMP tissues were mounted on slides, allowed to dry in total darkness, and then cover-slipped pending imaging.

#### Image acquisition and processing

An Olympus Fluoview F300 and a Zeiss LSM 110 laser scanning confocal microscope were used for image acquisition and processing. In dual-labeling experiments, a composite image targeting the two secondary conjugates was scanned simultaneously and were separately analyzed offline. The secondary antibody fluorophores for Alexa 488 was visualized using the 510-nm emission filter and the 605-nm filter for Alexa 594.

#### Comparison of packing density of β-arrestin2 expressing neurons in colon and ileum

Colocalization studies targeting β-arrestin2 and the neuronal marker HuC/D were performed to determine the packing density or percent of neurons expressing the β-arrestin2 in the colonic and ileum LMMP preparations. For the quantitative analysis, the anti-Hu C/D labeling was taken to represent the total number of neurons in the ganglia and the number of β-arrestin2 neurons was expressed as a percent of the Hu C/D positive cells.

#### Neurochemical coding of β-arrestin2 expressing neurons

To determine the chemical coding of β-arrestin2 expressing neurons, double labeling was performed using neurotransmitter markers (choline acetyltransferase, substance P and nitric oxide synthase) or calcium binding proteins (calbindin or calretinin). Colocalization between the β-arrestin2 and MOR_1_ receptor was determined by a modified method combining both fluorescence *in situ* hybridization for MOR_1_ and immunohistochemistry for the β-arrestin2.The primary and secondary antibodies used are outlined in table 1. The distribution pattern and extent of colocalization of β-arrestin2 positive neurons was assessed qualitatively. Cells were considered to be immunopositive if they expressed visually detectable labeling. Flouview software was used to capture the images and Image J software was used to analyze the distribution and determine the density of immunopositive neurons.

**Table 1:** List of antibodies for Immunohistochemistry.

Antibody (Host)	Source	Dilution
*Primary antibodies*		
β-arrestin2 (rabbit)	Cell signaling	1∶150
Hu C/D (mouse)	Abcam	1∶50
Substance P (mouse)	Abcam	1∶1500
ChAT, acetyltransferase (goat)	Millipore	1∶50
Calretinin (goat)	Swant	1∶100
Calbindin (mouse)	Swant	1∶100
NOS (nitric oxide transferase)	Santa Cruz.	1∶50
*Secondary antibodies*		
Alexa 488-conjugate	Life Technologies	1∶500
Alexa 594-conjugate	Life Technologies	1∶500

### Functional assessment of β-arrestin2 deletion on longitudinal muscle contraction

Following euthanization by cervical dislocation, the segments of ileum and colon were dissected, flushed of their contents, and trimmed of mesentery. The tissues, 1 cm in length, were suspended vertically along the axis of the longitudinal muscle and tied at each end with a fine thread. The thread was then passed through platinum-ring electrodes and placed in an organ bath containing Krebs buffer solution with one thread tied to a force transducer and the other fixed to a tissue holder under 1 g of passive tension. The tissues were allowed to equilibrate for 60 minutes prior to drug exposure, during which the Krebs solution was changed every 15 minutes. Isometric contractions were recorded by a force transducer (GR-FT03; Radnoti, Monrovia, CA) connected to a personal computer using Acknowledge 382 software (BIOPAC Systems, Inc., Santa Barbara, CA).

Following equilibration, the tissues were exposed to non-cumulatively increasing concentrations of nicotine (final concentrations in the organ bath ranging between 0.1 µM and 100 µM). Three 4-minute washes followed by a single 10 minute wash with drug free Krebs solution were performed between each drug challenge to permit recovery of the tissue. In each experiment, two LMMP preparations from each test group of animals were studied simultaneously and the responses of the tissues from the same animal averaged. The effect of each agonist on the amplitude of the neurogenic contractions was calculated as percent stimulation from the reference acetylcholine-induced (3 µM) contractions measured at the end of the experiments. The values were used to determine the EC_50_ (i.e. concentration required to produce 50% of the maximum response) and to calculate the maximum tension produced by nicotine. Computer assisted analysis of each concentration-response curve using Graphpad Prism software (SPSS Inc.) was employed to determine the EC_50_.

### Data analysis

All data was analyzed using appropriate statistical tools using the GraphPad Prism software (GraphPad Software Inc.). Analysis was performed to compare the density of neurons expressing β-arrestin2 (i.e. neurons positive for β-arrestin2/neurons positive for Hu C/D) between the ileum and colonic tissues. Significant differences between the test and control groups were determined using unpaired Student's “t” test. Immunofluorescence images were analyzed qualitatively to determine colocalization between β-arrestin2 and the targeted proteins or MOR_1_ RNA. For nicotine stimulation experiments, the EC_50_ and the maximal isometric tension produced were determined and compared. The percent contraction was calculated using the mean contraction height divided by the maximal contraction induced by acetylcholine (3.0 µM). Contractile responses after repeated administrations of the agonist were analyzed by repeated measures ANOVA followed by Bonferroni post hoc test. Data are presented as mean ± S.E.M. Values of P<0.05 were considered to be statistically significant.

## Results

### Expression and density of β-arrestin2 in the LMMP

**Figure 1:**
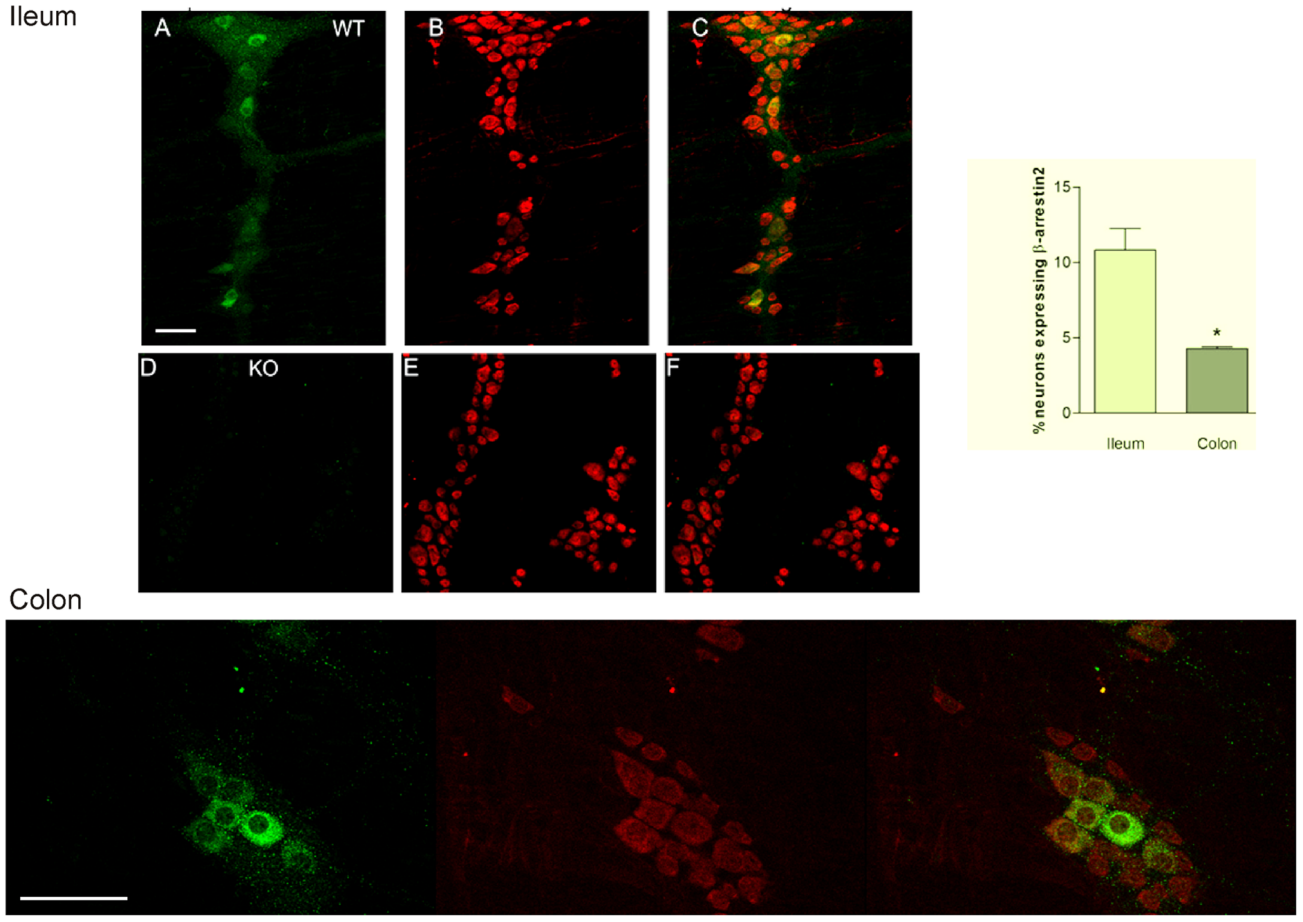
Expression of β-arrestin2 in the myenteric plexus. β-arrestin2 is exclusively expressed in a select group of neurons in the myenteric ganglia. Typical images from the longitudinal muscle/myenteric preparation showing immunofluorescence in neurons expressing β-arrestin2 - green and Hu C/D - red in wild type (A, B, C) and β-arrestin2 KO (D, E, F); note absence of β-arrestin2 staining in β-arrestin2 KO. Comparison of the density of β-arrestin2 expressing neurons in colon and ileum (bar graph on right). The density of β-arrestin2 immunopositive neurons was higher in the ileum (10.82%) compared to the colon (4.27%), *p≤0.05. Bottom panel are high magnification scans of β-arrestin2 and Hu C/D in myenteric ganglia from mouse colon. Scale bars: Upper panels: 20 µm, lower panel 200 µm.

Initial studies, using the whole mount (LMMP) were performed, to verify the specificity of the anti- β-arrestin2 primary antibody and to determine the spatial expression of β-arrestin2 within the myenteric ganglia from C57 BL/6 wild type and β-arrestin2 knockout mice. Fig. 1 illustrates confocal scans of the whole mount with green immunofluorescence depicting neurons expressing β-arrestin2 and red showing Hu C/D immunopositive neurons in wild type mice (A, B, C). Prototypical images from wild type mice show distinct and exclusive expression of β-arrestin2 in a select group of neurons in the myenteric ganglia from the ileum (top panel) and in the colon (bottom panel). The expression of β-arrestin2 appeared to be mostly confined to the soma and little was observed in the axonal or dendritic processes. β-arrestin2 expression was clearly absent within the nuclear compartment. The neurons expressing β-arrestin2 were morphologically homogeneous with relatively larger cell bodies that are ovoid and slightly elongated compared to the smaller and circular shaped neurons that were only positive for the Hu C/D marker. Comparison of the immunofluorescence signal within ganglionic neurons and the underlying longitudinal muscle shows little or no β-arrestin2 expression in the smooth muscle layer. No distinct immunofluorescence was evident for β-arrestin2 in the whole mount of β-arrestin2 KO mice except for the non-specific background staining (Fig. 1D, E, F). The slightly faint immunofluorescence observed in the underlying longitudinal muscle could either be background staining or show that this protein is not significantly expressed in the muscle. In some of the mounts, we observed faint immunofluorescence in the inter-ganglionic connections, varicosities and tertiary neuronal processes.

To compare the relative density of the neurons expressing β-arrestin2 in the colon and ileum of C57 BL/6 wild type mice, dual labeling was performed using antibodies targeting β-arrestin2 and HuC/D, a marker used to represent the total number of neurons in the ganglia. Bar graph in Fig. 1 shows a comparison of the density of β-arrestin2 expressing neurons in colon versus the ileum. Interestingly, the density of β-arrestin2 immunopositive neurons was significantly higher in the ileum (10.82%±1.4) compared to the colon (4.27%±0.14). The significance of this variance is not yet apparent and will be investigated in additional studies.

**Figure 2:**
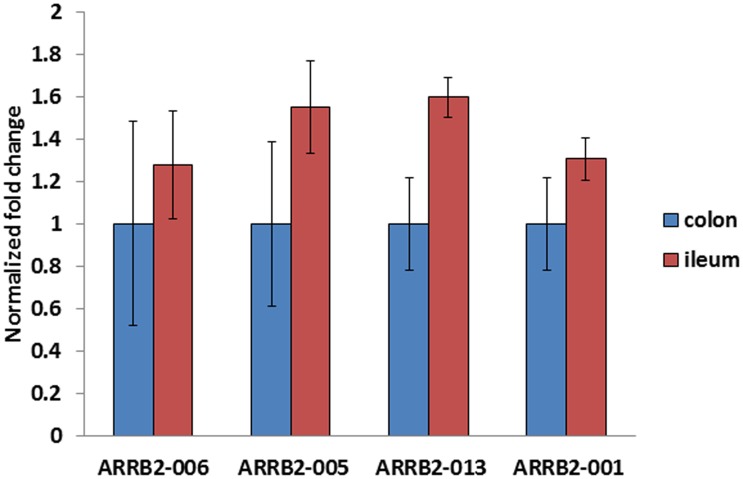
Expression of β-arrestin2 variants in Ileum. Real time PCR analysis depicts presence of four β- arrestin2 variants in ileum and colon. The relative expression of each variant was examined in the ileum and colon (n = 3) with 18s rRna was used as an endogenous control.

β- arrestin 2 is known to have 13 splice variants of which six produce functional proteins. We have previously reported that β-arrestin levels in the ileum decrease in response to chronic morphine exposure that results in the induction of tolerance. Thus, it is important to know which transcripts are expressed in the ileum. However, there are no previous reports to elucidate the nature of the transcripts that are encoded in the ileum and colon. A preliminary mi-SEQ analysis (data not shown) identified the presence of four of the thirteen variants. We confirmed that ARBB2-005, ARBB2-006, ARBB2-013, and ARBB2-001 transcripts are found in the ileum and in the colon (figure 2). These transcripts are reported to encode for proteins that are similar to isoforms a and b of β- arrestin2. The expression of each of the transcripts was greater in ileum compared to the colon.

### Neurochemical coding of neurons expressing â-arrestin2

#### Neurotransmitter markers

**Figure 3:**
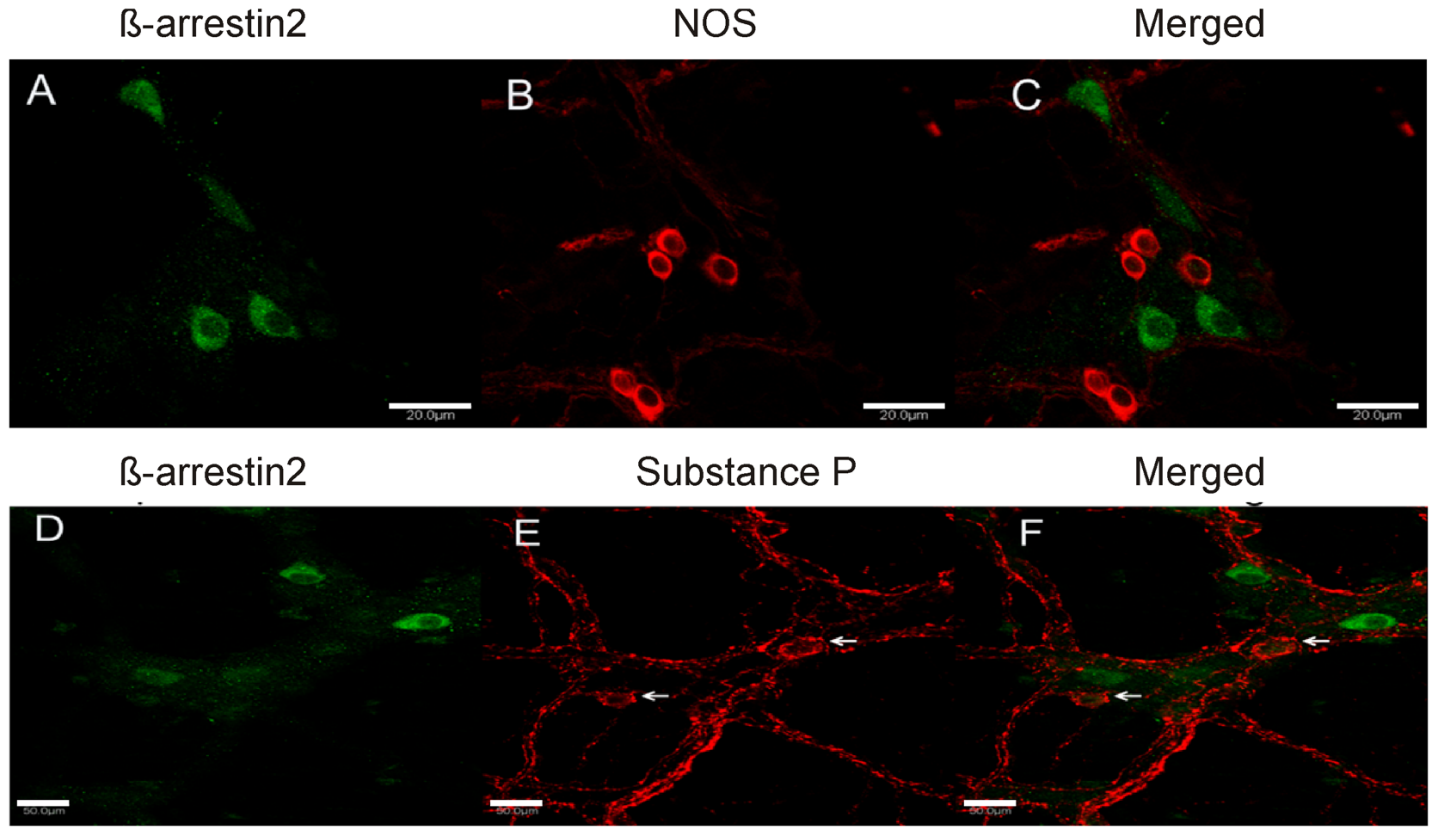
Representative images of LMMP whole mounts comparing expression of NOS, and substance P in β-arrestin2 immunopositive neurons. **A**: β-arrestin2-immunopositive neurons (green). **B**: NOS -immunopositive neurons (red). **C**: merged image depicting of β-arrestin2 and NOS fluorescence. **D**: β-arrestin2-immunopositive neurons (green). **E**: Substance P-immunopositive neurons (red). **F**: merged image depicting colocalization of β-arrestin2 and Substance P. Scale bars: Upper panel 20 µm: bottom panel 50 µm.

**Figure 4:**
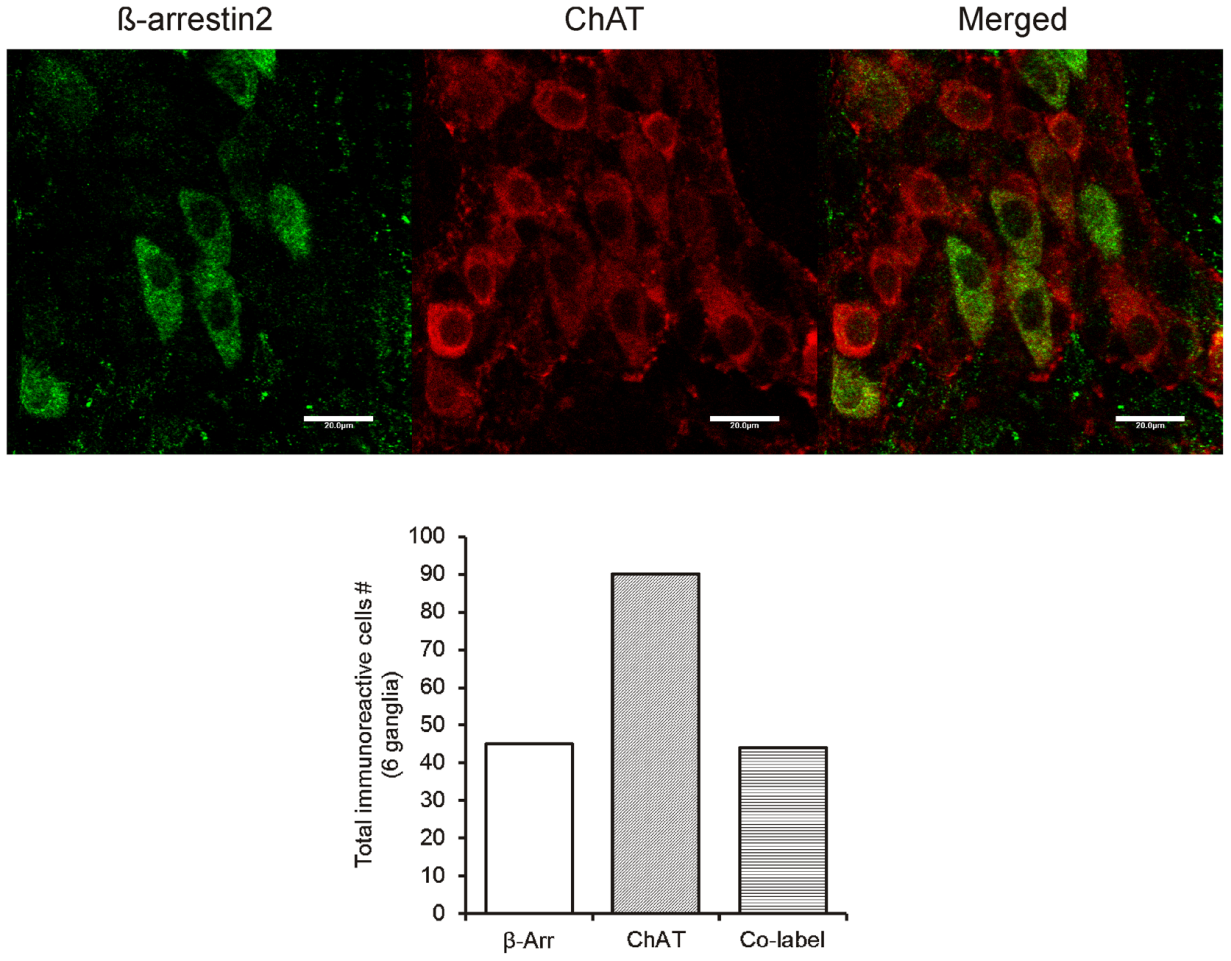
β-arrestin2-immunopositive neurons (green) and ChAT -immunopositive neurons (red), merged image depicting co-localization of β-arrestin2 and ChAT (yellow). Bottom bar graph are total β-arrestin2 positive and ChAT positive neurons from 6 ganglia. Scale bars: 20 µm.

Previous studies focusing on the myenteric ganglia have shown that the majority of excitatory neurons are associated with acetylcholine and substance P whereas inhibitory neurons tend to express nitric oxide (NO), vasoactive intestinal peptide (VIP), norepinephrine, adenosine triphosphate (ATP) and pituitary adenylate cyclase-activating polypeptide (PACAP) (McConalogue and Furness, 1994). To determine the neurotransmitter coding of neurons expressing β-arrestin2 we utilized markers targeting ChAT, nitric oxide synthase (NOS) and substance P which are markers for cholinergic, nitrergic and tachykinergic neurons respectively. Representative images from whole mounts of the mouse LMMP showing the neurotransmitter markers in the myenteric plexus are shown in Fig 3 and 4. The green immunofluorescence (Fig 3 A and D) indicating β-arrestin2-expressing neurons is consistent with previous findings with respect to morphological structure and distribution. The red immunofluorescence in Fig 3B depicts widespread NOS expression in the myenteric ganglia, however, no co-localization is observed with β-arrestin2 suggesting that β-arrestin2 is not expressed in inhibitory nitrergic neurons. Substance P is a critical moiety involved in excitatory signaling in the enteric nervous system and has been reported to be co-released with acetylcholine. We therefore assessed whether β-arrestin2 is also co-localized with substance P. To enhance substance P immunofluorescence signal within the soma, tissues were incubated in colchicine for 4 hours prior to fixing with paraformaldehyde. Following colchicine treatment, substance P pattern of distribution was not only visible in cell bodies but also in varicosities and tertiary neuronal fibers (Fig. 3E). The merged images depicting β-arrestin2 and substance P expression (Fig. 3F) clearly show that the two proteins are expressed in different neurons since no co-localization was observed. The red immunofluorescence in Fig 4 shows ChAT expressing neurons; upon merging with β-arrestin2 expression, it is evident that robust expression of β-arrestin2 was observed in cholinergic neurons as depicted by the cell bodies in merged image. The majority of β-arrestin positive cell bodies co-localized with ChAT which accounted for about 50% of all ChAT positive neurons in 6 separate ganglia (Fig 4).

**Figure 5:**
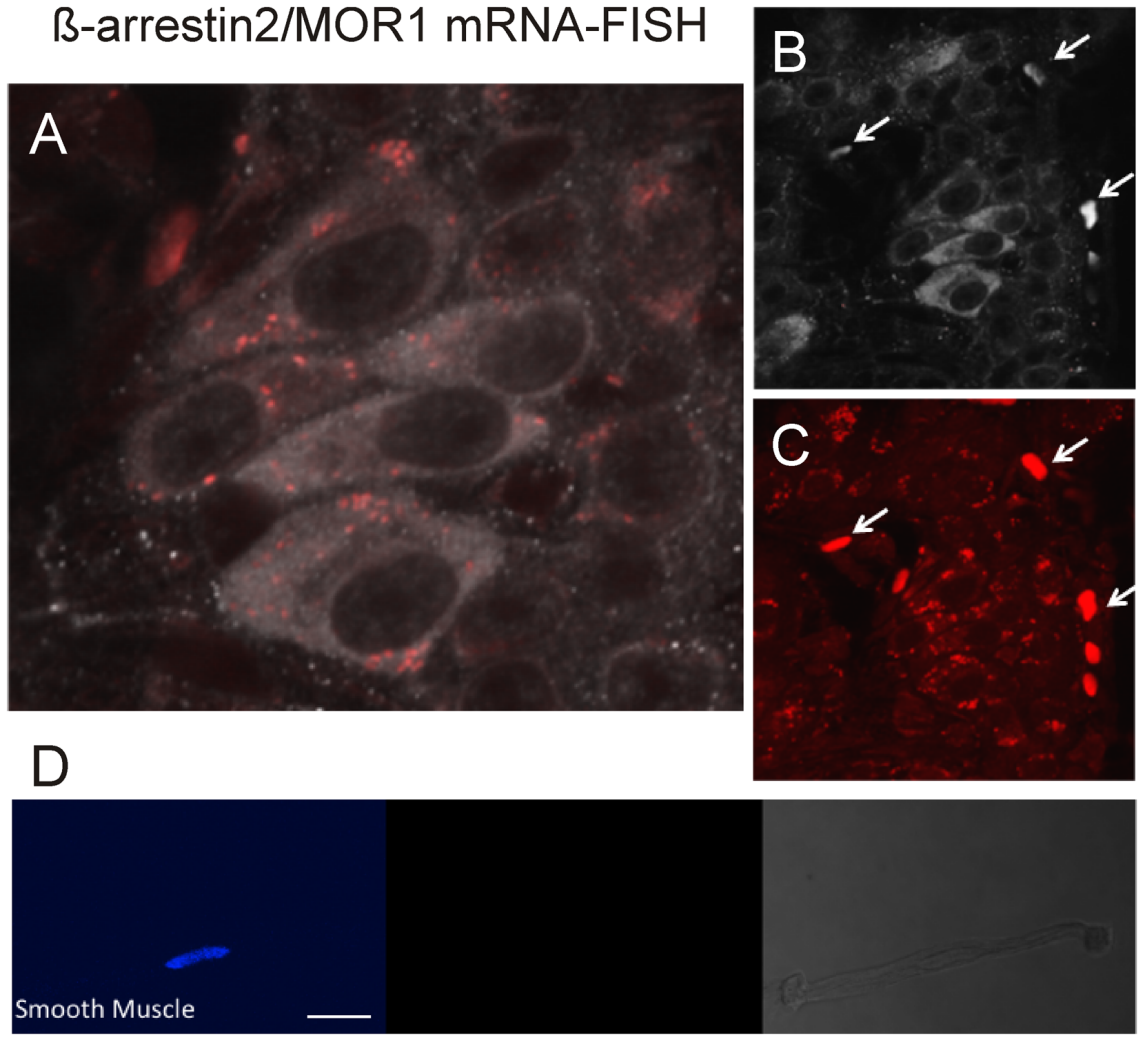
Image of myenteric ganglia expression of MOR_1_ mRNA in β-arrestin2 immunopositive neurons. β-arrestin2-immunopositive neurons (grey) with punctate mRNA for MOR_1_ (red) determined my mRNA-FISH. A) High magnification of merged image showing expression of MOR1 (red) within cell bodies labeled with β-arrestin2 (grey). B) grey scale image of β-arrestin2, C) fluorescent Cy5 labeled MOR1 – mRNA. Arrows are non-specific staining of cells outside the ganglia. D) Cy5 labelling of MOR1 mRNA in isolated ileum smooth muscle cell. Nuclear stain (blue). Middle panel indicates the absence of Cy5 fluorescent in smooth muscle. Bright field image of smooth muscle cell on the right panel. Scale bar: 20 µm.

We investigated the expression of MOR1 in neurons. As suggested previously, the antibody to MOR1 shows significant non-specificity [17], we therefore examined expression of MOR1 transcript by mRNA-FISH. Based on Taylor and Fleming's criteria for assessing drug induced plasticity the changes observed must “*quantitatively account for the tolerance*” and “*occur in the very cells upon which the opioid is acting*” [10], we assessed whether β-arrestin2 was indeed co-localized with the MOR_1_ receptor. Fig. 5 illustrates confocal scan of the whole mount with gray image depicting β-arrestin2 expression in cell bodies within the myenteric ganglia and red punctate fluorescence of the mRNA for MOR_1_ (Fig. 5). Consistent with mRNA expression, the MOR_1_ receptor expression, represented by defined punctate staining, was clearly distinct in the cytoplasmic space and none in the nuclear compartment. Control experiments using underlying longitudinal muscle that is devoid of the myenteric ganglia show little or no MOR_1_ mRNA expression hence confirming validity of the preparation and specificity of the Cy5 labeled oligonucleotide probe. Furthermore, expression of mRNA-FISH for MOR1 was also not evident in isolated smooth muscle cells (fig 5D). Intense non-specific staining was observed in red blood cells (arrows).

#### Co-localization with calcium-binding proteins

**Figure 6:**
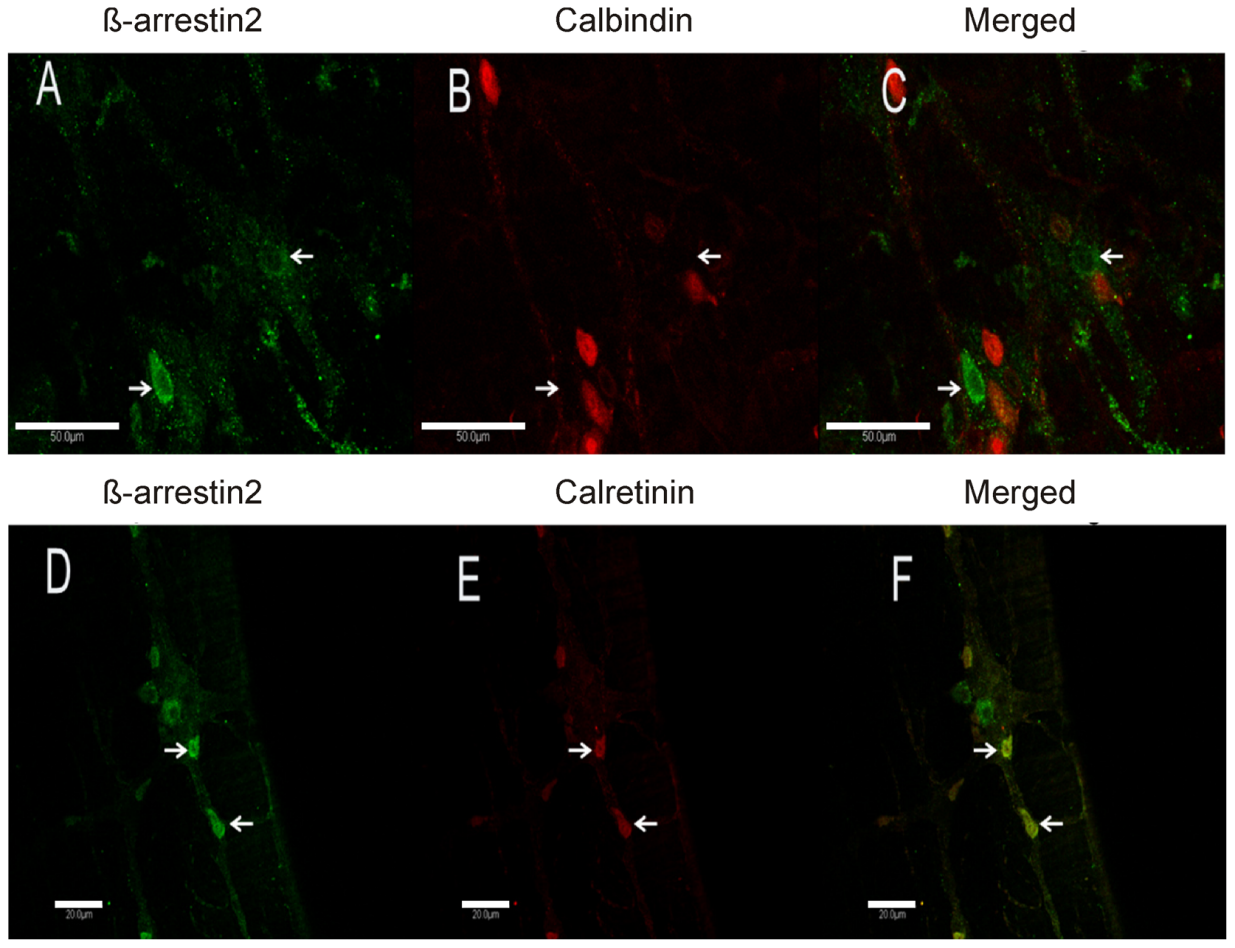
Representative images of LMMP whole mounts comparing expression of calbindin and calretinin in β-arrestin2 immunopositive neurons. **A**: β-arrestin2-immunopositive neurons (green). **B**: calbindin-immunopositive neurons (red). **C**: merged image depicting expression of β-arrestin2 and calbindin. **D**: β-arrestin2-immunopositive neurons (green). **E**: calretinin -immunopositive neurons (red). **F**: merged image depicting colocalization of β-arrestin2 and calretinin (yellow). Scale bars: Upper panel: 50 µm, bottom panel: 20 µm.

For these experiments, we utilized two calcium binding protein markers; calbindin, a conserved protein usually used to mark sensory neurons [15], and calretinin which has been widely used as a marker for motor neurons in guinea pigs [18]. Representative images from whole mounts of the mouse LMMP showing calbindin and calretinin immunopositive neurons in the myenteric plexus are provided in Fig 6. The green immunofluorescence (Fig 6A, D) indicates β-arrestin2-expressing neurons while the red immunofluorescence in Fig 6B depicts calbindin expressing neurons and the red in Fig 6E depicts calretinin expressing neurons. As shown in the merged images, β-arrestin2 is clearly not expressed in calbindin-positive neurons. Calbindin expression was observed in the soma and axonal projections. The multiple dendritic projections are consistent with the observations reported in guinea pigs and correlate with Dogiel type II neuronal structure of intrinsic primary sensory afferent neurons [18]. In addition, the varicosities seem to reside predominately in the periphery of the myenteric ganglia whereas the β-arrestin2 expressing cell bodies are congregated in the central part of the ganglia. In contrast, robust expression of β-arrestin2 was observed in calretinin immunopositive neuron as depicted by the yellow cell bodies (Fig 6F). Table 2 is a summary of the results of neurochemical coding for a proteins assessed. In summary, β–arrestin 2 was found in calretinin expressing neurons and not in Calbindin expressing neurons.

**Table 2:** Chemoarchitecture of β-arrestin2 expressing myenteric neurons.

Type of neuron	Marker	Result
Nitrergic	Nitric oxide synthase	-
	Calbindin	-
Cholinergic	Choline acetyltransferase (ChAT)	+
	Calretinin	+
Tachykinergic	Substance P	-

### Effect of β-arrestin2 deletion on the sensitivity to nicotine

**Figure 7:**
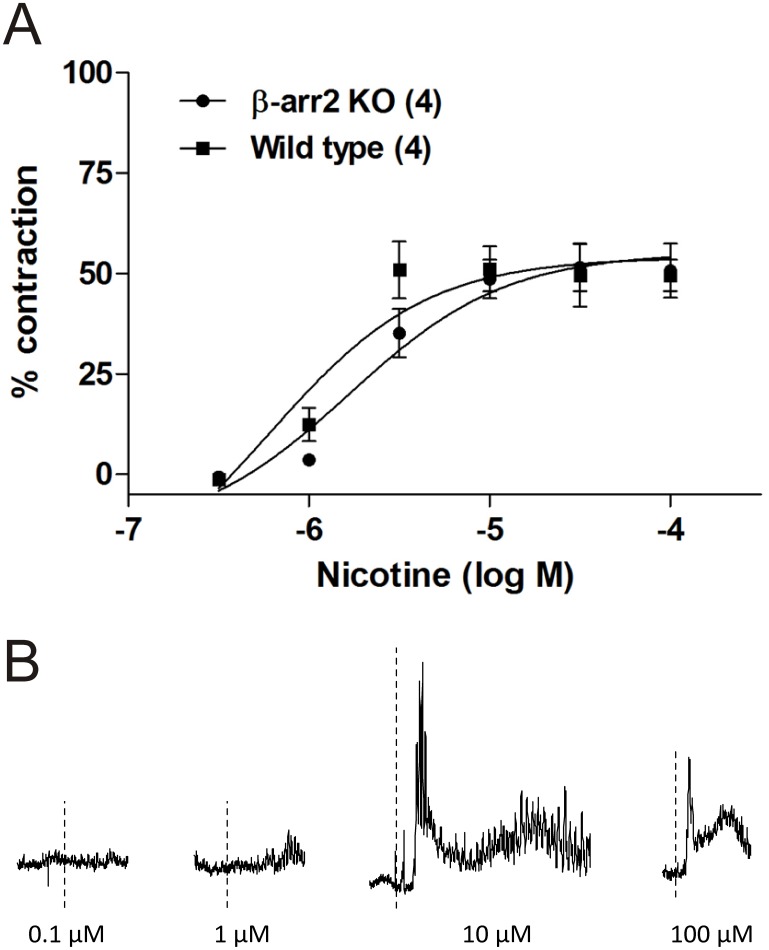
Mean concentration-response curves for nicotine in longitudinal ileum preparation obtained from WT or β-arrestin2 KO mice. A slight but insignificant change to the sensitivity to nicotine was observed in the β-arrestin2 knockout mice (A). B) representative isometric tension recording tracing of the longitudinal ileum preparation from C57BL/6 WT mice showing the non-cumulative dose response effect of repeated exposure with nicotine. Statistically significant differences (p≤0.05) are identified by *.

**Figure 8:**
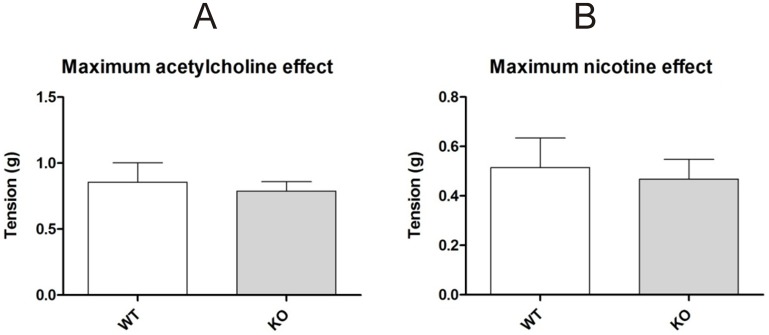
Shows a bar graph comparing the maximal tension values attained by nicotine (A) or acetylcholine (B) stimulation in the longitudinal ileum preparation from WT or β-arrestin2 KO mice. No significant increase in maximal efficacy to nicotine or acetylcholine. Statistically significant differences (p≤0.05) are identified by *.

One of the issues associated with using genetic knockouts is the possibility of an aberrant change in normal physiological function. Based on the previously outlined results that show clear expression of β-arrestin2 in the myenteric ganglionic cholinergic neurons, we set out to determine whether genetic deletion of this protein would alter basal physiological cholinergic transmission in the myenteric ganglia. To this end, we used the longitudinal muscle preparation from C57 BL/6 wild type versus the paired β-arrestin2 knockout mice to assess possible changes in potency and efficacy of nicotine which targets the neuronal nicotinic receptors exclusively expressed in the myenteric plexus. As illustrated in Fig. 7, genetic deletion of β-arrestin2 did not change the functional response of the ileum longitudinal muscle to nicotine since the potency was similar among the two groups (-log EC50: C57BL/6 wild type = 5.76 vs. β-arrestin2 knockout = 5.90). A marginal decrease in the maximal efficacy of nicotine was observed (Fig. 8) but it was not statistically significant (C57BL/6 wild type = 0.67 g±0.18 vs. β-arrestin2 knockout = 0.44 g±0.07; P = 0.26).

## Discussion

Previous studies from our lab strongly suggest a direct interaction between MOR activation and β-arrestin2 since downregulation of β-arrestin2 is associated with the development of tolerance to opioids in the intestinal tract [8] [7]. β-arrestin2, is a key molecule involved in desensitization of activated G protein-coupled receptors. We therefore examined possible colocalization of β-arrestin2 with the MOR_1_ receptor. In the present study, we sought determine the relative expression of β–arrrestin2 in various portions of the gastrointestinal tract as well as intracellularly in these tissues. Further, we determined the nature of the β- arrestin2 isoforms expressed in ileum and also the type of enteric cells expressing β-arrestin2. We investigated the impact of β-arrestin2 abrogation on basal cholinergic function. A number of studies have focused on studying the spatial expression of β-arrestin2 in the CNS, but few have investigated the specific localization of β-arrestin2 in the enteric nervous system.

β-arrestin2 is not only required for desensitization of the activated GPCR, but also serves as a signaling moiety through Janus kinase (JNK), mitogen activated kinase (MAK) and Src kinase pathways [19]-[21]. Because of its integral function in cell plasticity it is critical to assess chemoarchitecture of neurons expressing β-arrestin2 in the myenteric ganglia. To the best of our knowledge, this is the first study undertaken to determine the nature and spatial expression of β-arrestin2 within the myenteric ganglia. We report the presence of ARBB2-005, ARBB2-006, ARBB2-013 and ARBB2-001 transcripts in the ileum. These transcripts encoded for protein that are similar to the reported β-arrestin2 isoform a and isoform b proteins. The distribution of individual variants show that ARBB2-005 is the most abundant transcript followed by ARRB2-013. This study for the first time unravels the identity of the β- arrestin variants in ileum and colon. Understanding the function of these proteins in both the ileum and brain as opposed to the colon might well be useful information in studying the differences in morphine tolerance in these tissues. The primary findings of this study describe the presence of β-arrestin2 in neuronal cell bodies of both tissues, but do not distinguish the role of this protein in opioid tolerance. Further studies to examine the changes in expression levels will be required to elucidate the functional role in tolerance development.

Our studies also demonstrate selective expression of β-arrestin2 in the enteric nervous system in contrast to the ubiquitous expression of this protein in the CNS [5]. The β-arrestin2 expressing neurons are remarkably distinct. They are comparatively larger, more oval-shaped and can be clearly distinguished from the smaller and circular NOS expressing neurons [22]. The colocalization of β-arrestin2 with ChAT and calretinin suggests that these neurons are cholinergic and may likely be motor neurons. However, it should be noted that distinction of motor and sensory neurons based on the calcium-binding proteins has been defined in the guinea-pig but may differ in mice. Since a fraction of the β-arrestin2 positive neurons did not co-express ChAT, suggests that the β-arrestin2 expressing neurons are neurochemically heterogeneous. Other possible neuromarkers that could be utilized to further characterize the neurons include serotonin, VIP or PACAP.

The density of neurons expressing β-arrestin2 is significantly higher in the ileum compared to the colon. While the significance for this physiological difference is not apparent, it could have a bearing on the differences in GPCR plasticity and the level of morphine tolerance seen in the two segments of the gastrointestinal tract [8]. The fact that we discovered selective expression of β-arrestin2 in enteric neurons challenges the notion that this protein is ubiquitously expressed in cells that express GPCRs. Several GPCRs including the M_2_ muscarinic receptors are expressed in enteric smooth muscle cells. In the present studies we show that β-arrestin2 is not found in these cells suggesting that other β-arrestin2-independent pathways may dominate GPCR desensitization.

In previous studies, we have shown that the development of tolerance to morphine in the isolated ileum is associated with a time dependent reduction in the β-arrestin2 levels [8]. However, in those studies, western immunoblotting in digested isolated myenteric neurons or LMMP tissue was used to assess protein expression. Unfortunately, this method neither reveals the spatial location of the cells expressing β-arrestin2, morphological structure of the cells nor possible neuronal projections. More importantly, no direct link could be established between cells being activated by morphine and those where β-arrestin2 is expressed. The current study reports that the majority of neurons expressing β-arrestin2 also expressed MOR_1_ suggesting a possible direct intracellular link between MOR_1_ activation and β-arrestin2 downregulation associated with opioid tolerance in the ENS. In addition, the selective expression of MOR_1_ in neurons confirms our previous functional findings and support the school of thought that MOR_1_ receptors are selectively expressed in the neurons and not in the smooth muscle. Our discovery that β-arrestin2 is exclusively expressed in neuronal cells suggests that the opioid tolerance observed following β-arrestin2 deletion is likely due to plasticity within the myenteric plexus [7]. Based on the current findings showing co-localization between MOR_1_ and β-arrestin2, and on previously published data showing morphine induced downregulation of β-arrestin2 [5], [8], we postulate that morphine tolerance triggered by sustained activation of MOR_1_ receptors is, at least partially, imparted in β-arrestin2 expressing neurons and potentially represents an important therapeutic target.

Though most studies have utilized the guinea pig LMMP model to characterize myenteric neuron identity, one major advantage of using the murine model is the availability of genetic knockouts. In the current study, we were able to classify the neurons using five specific markers. However, we had difficulty characterizing the neurons morphologically according to the well-established Dogiel and Brehmer classifications which distinguish neurons based on the number and length of axonal and dendritic projections [12]. Our experiments using the neurofilament marker (results not shown) were especially challenging and complicated by the fact that axonal and dendritic projections in the murine LMMP are not as elaborate as other species e.g. guinea pig hence makes it difficult to distinguish the subtypes of mono-axonal neurons [16].

One drawback of utilizing genetic knockout models is the possible aberration of a normal physiological response as has been frequently observed in several models. The current study clearly shows that the abrogation of β-arrestin2 did not alter the efficacy and potency of ganglionic cholinergic innervation in the isolated longitudinal preparation. This finding supports our previous observation in the isolated circular muscle [7] and suggests that β-arrestin2 is not involved in the acute effects of cholinergic neuronal activation. Alternatively, since the cholinergic system is so expansive in the myenteric ganglia, the organ bath assay may not have been ideal to discern the subtle changes imparted by β-arrestin2 deletion in neurons since these constitute 10% in the ileum and only 4% in the colon. We are in the process of investigating the effect of chronic opioid exposure on the potency of agents targeting the nicotinic ganglionic receptors.

In summary, the data presented in this paper show that β-arrestin2 is expressed in myenteric neurons and not other cell types like smooth muscles or glia. In addition, MOR_1_ receptors appear to be co-expressed in a β-arrestin2 positive myenteric neurons that are likely cholinergic motor neurons. These neurons may be directly involved with the tolerance observed following chronic morphine exposure since it is associated with a time dependent reduction in β-arrestin2 levels within the myenteric plexus. The fact that β-arrestin2 is expressed in cholinergic neurons suggests a complex interplay between intraneuronal and possibly interneuronal interaction that may reflect the specific adaptive processes involved in the development of tolerance to opioids. The greater density of neurons expressing β-arrestin2 in the ileum compared to the colon might be a contributing factor to the expression of tolerance to opioids in the ileum and not the colon.
